# Mediastinal seminoma associated with multilocular thymic cyst

**DOI:** 10.1186/s40792-016-0278-7

**Published:** 2017-01-05

**Authors:** Masato Inui, Jun-ichi Nitadori, Shogo Tajima, Takahusa Yoshioka, Noriko Hiyama, Takeyuki Watadani, Aya Shinozaki-Ushiku, Kazuhiro Nagayama, Masaki Anraku, Masaaki Sato, Masashi Fukayama, Jun Nakajima

**Affiliations:** 1 Department of Thoracic Surgery, The University of Tokyo Graduate School of Medicine, Tokyo, Japan; 2 Department of Pathology, The University of Tokyo Graduate School of Medicine, Tokyo, Japan; 3 Department of Radiology, The University of Tokyo Graduate School of Medicine, Tokyo, Japan; 4 Department of Thoracic Surgery, School of Medicine, The University of Tokyo Hospital, 7-3-1 Hongo Bunkyo-ku, Tokyo, 113-8655 Japan

**Keywords:** Mediastinal tumor, Mediastinal seminoma, Multilocular thymic cyst

## Abstract

An asymptomatic 26-year-old man received an annual medical check-up, and chest X-ray showed a protrusion of the aortopulmonary window. Chest computed tomography (CT) revealed an anterior mediastinal tumor and cysts with thin wall and septum enhancement. The preoperative diagnosis was cystic thymoma or malignant lymphoma. We performed total resection of the tumor through a median sternotomy. The pathological findings revealed seminoma, positive for c-kit stain, and multilocular thymic cysts. Cysts were lined by normal squamous epithelium and no seminoma cells were located on their surface. So, cysts were probably secondary changes caused by seminoma cells themselves or inflammatory stimulations. No invasion to adjacent structures was seen. After the surgery, testicular ultrasound imaging and abdominal, pelvic, and cerebral CT showed no apparent tumor or enlarged lymph nodes; however, an abnormal uptake in the right mesenteric lymph node was pointed out by ^18^F-fluorodeoxyglucose-positron emission tomography (FDG-PET) scan. The patient received four courses of bleomycin, etoposide, and cisplatin (BEP) as adjuvant chemotherapy. Follow-up PET scan revealed no uptake in the right mesenteric lymph node. To date, no recurrence or metastasis has been identified for 16 months.

## Background

Prominent cystic change of mediastinal seminomas in patients is uncommon and rarely reported, and those cases that are reported show only unilocular cystic changes. Here, we present a patient with a mediastinal seminoma with multilocular thymic cysts. It is probable that primary seminoma caused the cyst formation of the thymus.

## Case presentation

**Fig. 1:**
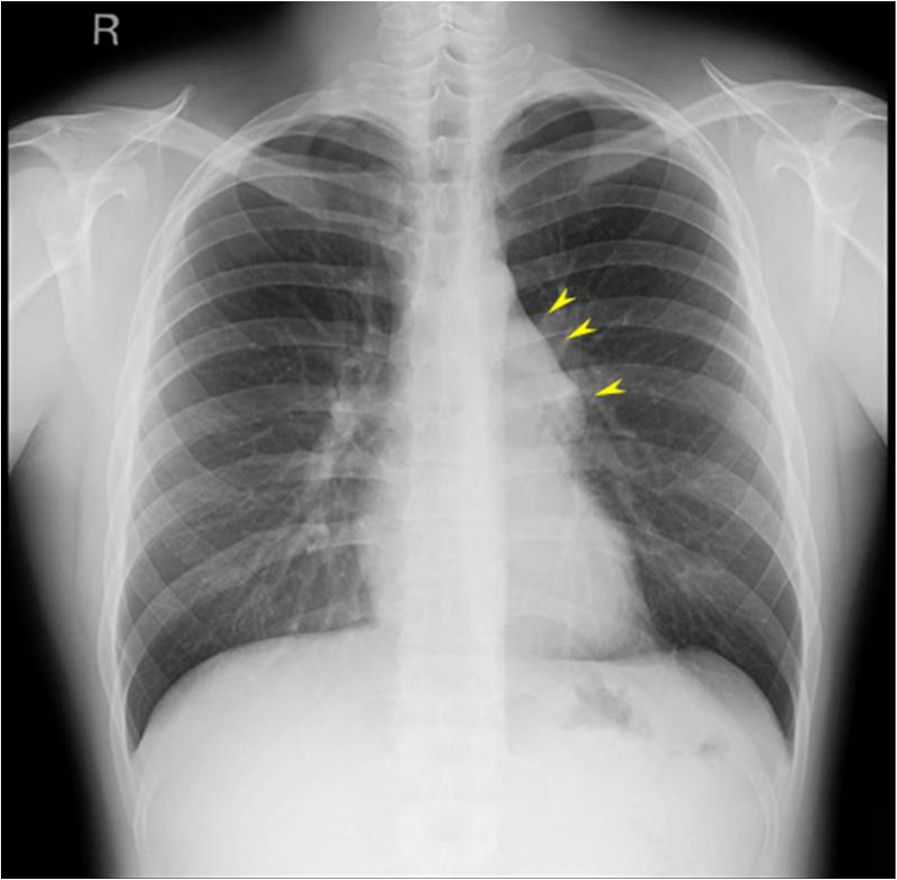
Chest X-ray showed a protrusion of the aortopulmonary window (*arrowheads*)

**Fig. 2:**
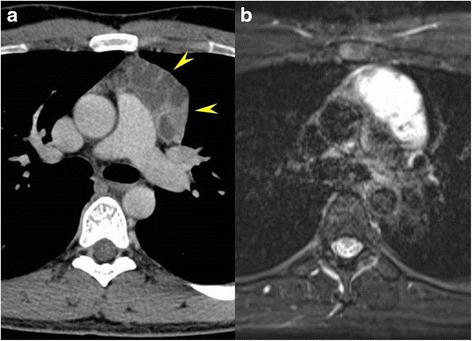
**a** Chest CT showing an anterior mediastinal tumor (6.3 cm × 3.6 cm × 9.0 cm, *arrowheads*) with multilocular cystic changes. **b** Fat-saturated T2-weighted MRI showing cystic lesions. No apparent invasion to the adjacent structures

**Fig. 3:**
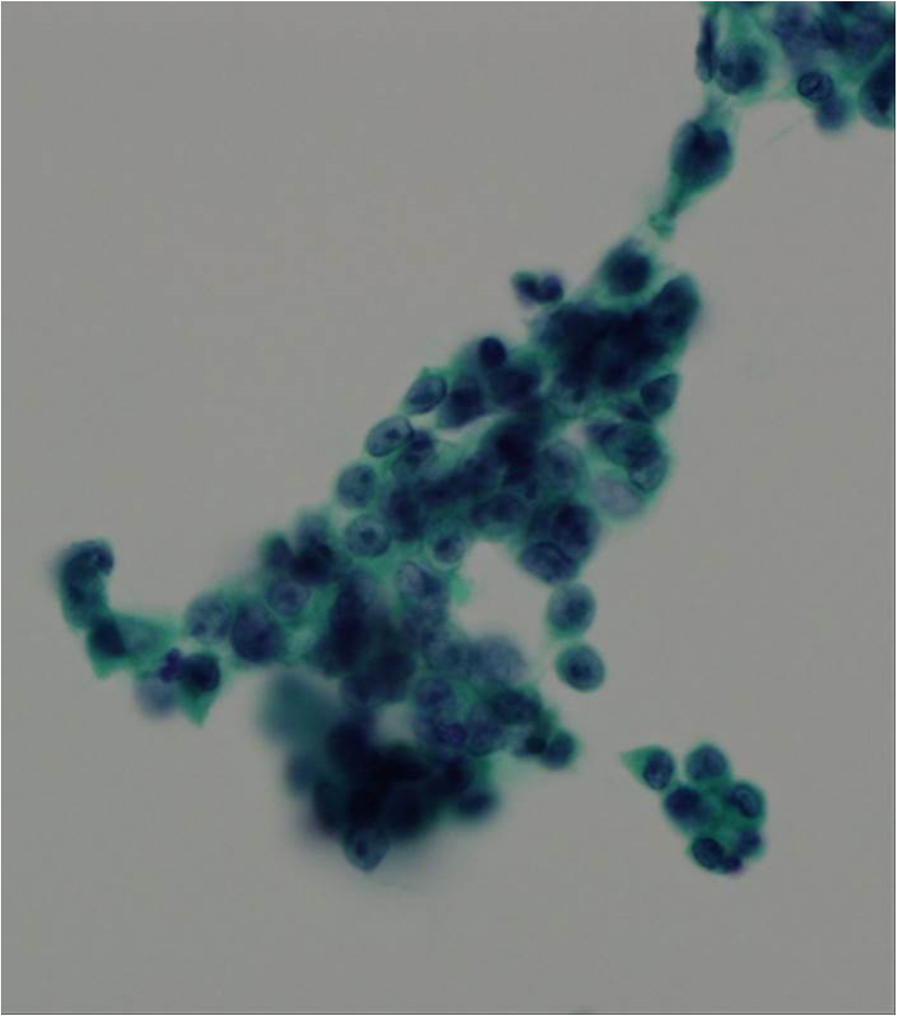
Cluster of seminoma cells from aspiration cytology. (Papanicolaou stain, ×600)

**Fig. 4:**
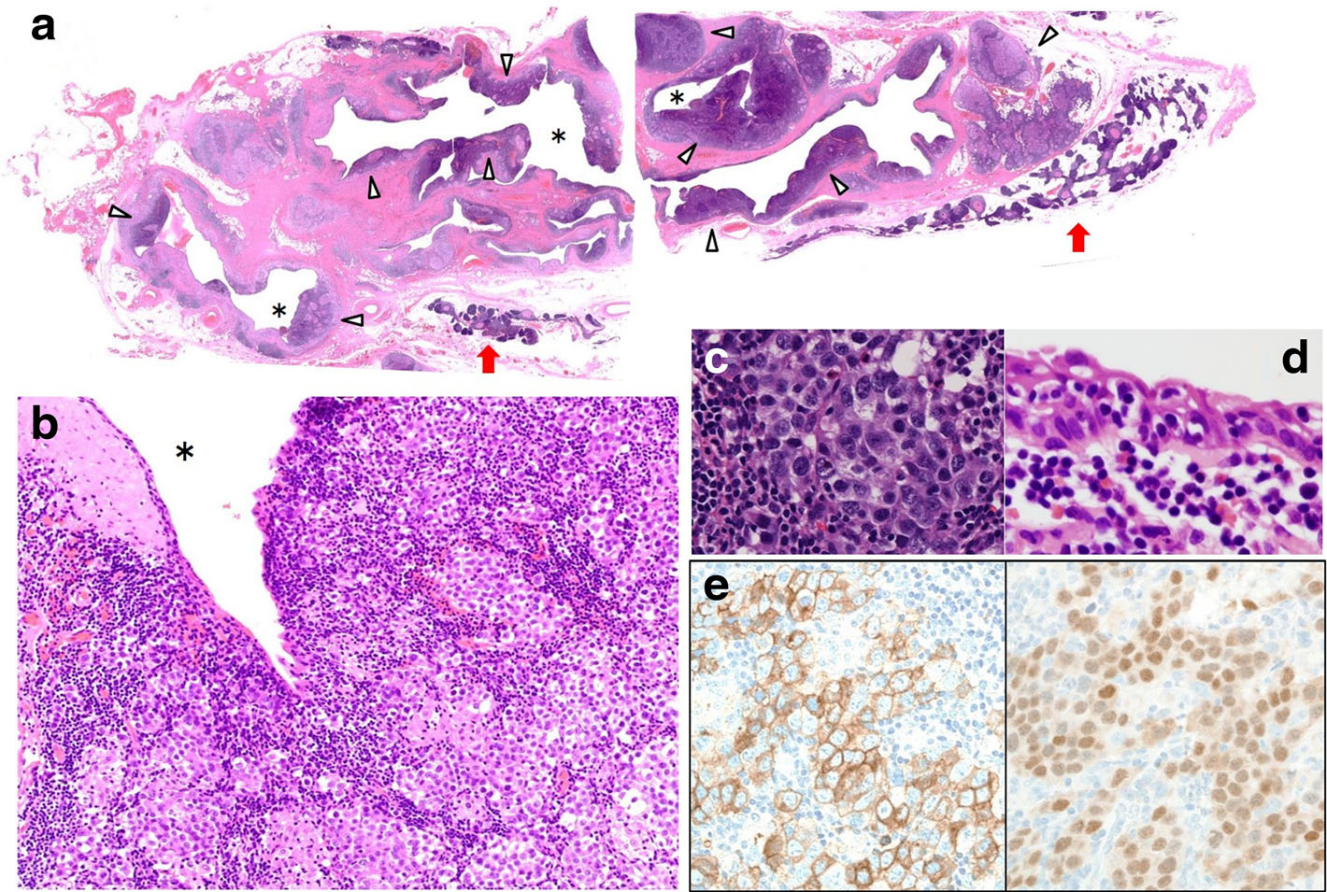
**a** Loupe view of the lesion demonstrates seminoma components (*arrowheads*) lying along multilocular cysts (*asterisks* indicate cystic space). *Arrows* indicate thymus (HE stain). **b** Close view of the cyst wall shows solid sheets of tumor cells with lymphocytic infiltrates. *Asterisk* indicates cystic space (HE stain, ×200). **c** Cluster of seminoma cells has oval nuclei with surrounding pale cytoplasm (HE stain, ×400). **d** Cysts (*asterisk*) were lined by nonneoplastic squamous epithelium, and no seminoma cells were located on the surface of cysts (HE stain, ×400). **e** Tumor cells are positive for PLAP (*left*) and OCT3/4 (*right*)

**Fig. 5:**
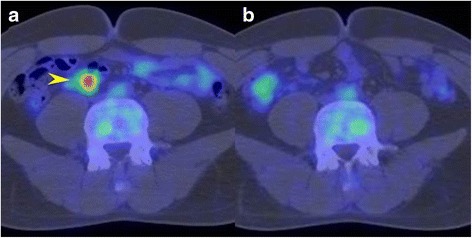
**a** FDG-PET revealed an abnormal uptake in a right mesenteric lymph node (*arrowhead*). **b** Follow-up PET showed disappearance of the abnormal uptake

A 26-year-old man underwent a chest X-ray for an annual check-up, which showed a protrusion of the aortopulmonary window (Fig. 1). Seven months later, he was referred to our hospital without symptoms. He had a past history of epilepsy with medication. He was a never smoker, but he was exposed to organic solvent due to his work as a researcher of carbon fiber. Chest CT revealed a thin-walled multilocular cystic tumor, 9.0 cm in diameter, in the anterior mediastinum. The septum of the tumor was enhanced by contrast medicine (Fig. 2a). The tumor compressed the left brachiocephalic vein, but seemed not to have invaded the surrounding structures. The serum level of carcinoembryonic antigen was slightly elevated (5.4 ng/ml; normal value, less than 5.0 ng/ml); however, other tumor markers including alfa-fetoprotein, beta-human chorionic gonadotropin, anti-acetylcholine receptor antibody, and soluble interleukin-2 receptor were not elevated. Magnetic resonance imaging (MRI) showed a multilocular cystic lesion with focal mural nodules in the anterior mediastinum (Fig. 2b). The preoperative diagnosis was a cystic thymoma, or a malignant lymphoma. We did not perform needle biopsy for diagnosis preoperatively, due to thin cystic wall. We performed a total resection of the tumor through a median sternotomy. The tumor was fully covered with fibrous capsule with partly dense adhesion to the left mediastinal pleura. Needle aspiration cytology of cystic fluid was done during surgery, and the result was class V (suspicion of seminoma) (Fig. 3). The left mediastinal pleura was resected with the tumor. The pathological diagnosis was seminoma with multilocular thymic cysts. Higher magnification showed large polygonal cells with clear cytoplasm, round or oval nuclei, and prominent nucleoli; immunohistochemical stain for c-kit, oct3/4, d2-40, and placental-like alkaline phosphatase stain was positive, and beta-human chorionic gonadotropin stain was negative [1]. So, these cells were diagnosed as seminoma cells. The cysts were lined by only normal squamous epithelium, and cyst walls were characterized by inflammatory changes (Fig. 4a–d). Testicular ultrasound imaging and abdominal, pelvic, and cerebral CTs did not show any apparent tumors or enlarged lymph nodes preoperatively. However, FDG-PET scan revealed an abnormal uptake (maximal standardized uptake value was 6.8) in the right mesenteric lymph node (Fig. 5a). The patient was classified as intermediate risk group due to suspicion of mesenteric lymph node metastasis, so received four courses of BEP (20 mg/m^2^ cisplatin on days 1–5, 100 mg/m^2^ etoposide on days 1–5, and 30kU bleomycin on days 1, 8, and 15, repeated 21 days) as adjuvant chemotherapy. Follow-up PET scan revealed no uptake in the right mesenteric lymph node (Fig. 5b). To date, no recurrence or metastasis has been identified for 16 months.

## Discussion

Mediastinal germ cell tumors account for only 1 to 5% of all germ cell tumors [2]. Seminomas account for only 0.4% of all mediastinal neoplasm in adults, and most are typically solid and lobulated in appearance [3, 4]. Only a few mediastinal seminomas showed prominent cystic changes, and most of those were unilocular and congenital. However, this case showed multilocular cystic changes, lined by normal squamous epithelium. So, these cysts were probably secondary ones caused by seminoma cells themselves or inflammatory stimulations. The pathogenesis of cystic changes in seminoma is still unclear but thought to be related to the irritation that tumor cells cause to Hassall corpuscles [5]. The process has been observed frequently as a secondary event complicated with a variety of thymic neoplasms, including thymoma, non-Hodgkin’s lymphoma, Hodgkin’s disease, and germ cell tumor (mainly mature cystic teratoma) [6, 7]. Initial management of primary mediastinal seminoma is cisplatin-based chemotherapy [8]; however, because of its rarity, some cases are diagnosed only after surgical resection. Fine needle aspiration cytology occasionally helps the preoperative diagnosis [9]. In this case, however, cyst walls were thin and solid lesions were located behind the left lung, so it was thought to be difficult to perform. According to International Germ-Cell Cancer Collaborative Group risk classification, the patient is classified as intermediate risk group to undergo chemotherapy. Four courses of BEP were administered as chemotherapy, which will result in a more favorable prognosis [10, 11]. When an anterior mediastinal tumor with multilocular cystic changes is observed, primary seminoma also should be listed as a differential diagnosis.

## Conclusions

Primary mediastinal seminoma should be listed as one of the differential diagnoses, when anterior mediastinal tumor with unilocular cyst is confirmed, especially the patient is young male.

## References

[1] Liu A, Cheng L, Du J (2010). Diagnostic utility of novel stem cell markers SALL4, OCT4, NANOG, SOX2, UTF1, and TCL1 in primary mediastinal germ cell tumors. Am J Surg Pathol.

[2] Hainsworth JD (2002). Diagnosis, staging, and clinical characteristics of the patient with mediastinal germ cell carcinoma. Chest Surg Clin N Am.

[3] Dulmet EM, Macchiarini P, Suc B (1993). Germ cell tumors of the mediastinum: a 30-year experience. Cancer.

[4] Strollo DC, Rosado-de-Christenson ML (2002). Primary mediastinal malignant germ cell neoplasmas: imaging features. Chest Surg Clin N Am.

[5] Weissferdt A, Moran CA (2015). Mediastinal seminoma with florid follicular lymphoid hyperplasia: a clinicopathological and immunohistochemical study of six cases. Virchows Arch.

[6] Suster S, Rosai J (1992). Cystic thymomas: a clinicopathologic study of ten cases. Cancer.

[7] Kim JH, Goo JM, Lee HJ (2003). Cystic tumors in the anterior mediastinum: radiologic-pathological correlation. J Comput Assist Tomogr.

[8] Fizazi K, Culine S, Droz JP (1998). Initial management of primary mediastinal seminoma: radiotherapy of cisplatin-based chemotherapy?. Eur J Cancer.

[9] Silverman JF, Olson PR, Dabbs DJ, Landreneau R (1999). Fine-needle aspiration cytology of a mediastinal seminoma associated with multilocular thymic cyst. Diagn Cytopathol.

[10] International Germ Cell Consensus Classification: a prognostic factor-based staging system for metastatic germ cell cancers. International Germ Cell Cancer Collaborative Group. J Clin Oncol. 1997;15:594-603. 10.1200/JCO.1997.15.2.594 9053482

[11] Testicular cancer treatment guidelines of Japanese urological association. 2015.

